# Proposal for a New Classification of Rare Cancers Adopting Updated Histological Tumor Types

**DOI:** 10.1111/pin.70021

**Published:** 2025-05-16

**Authors:** Ryoko Rikitake, Yasushi Yatabe, Yoko Yamamoto, Tatsunori Shimoi, Shintaro Iwata, Yasushi Goto, Yu Mizushima, Akira Kawai, Takahiro Higashi

**Affiliations:** ^1^ Division of Health Services Research Institute for Cancer Control, National Cancer Center Tokyo Japan; ^2^ Rare Cancer Center, National Cancer Center Hospital Tokyo Japan; ^3^ Department of Public Health and Health Policy Graduate School of Medicine, The University of Tokyo Tokyo Japan; ^4^ Department of Diagnostic Pathology National Cancer Center Hospital Tokyo Japan; ^5^ Department of Medical Oncology National Cancer Center Hospital Tokyo Japan; ^6^ Department of Musculoskeletal Oncology and Rehabilitation Medicine National Cancer Center Hospital Tokyo Japan; ^7^ Department of Thoracic Oncology National Cancer Center Hospital Tokyo Japan

**Keywords:** cancer classification, International Classification of Diseases, National Cancer Registry, rare cancer, Surveillance of Rare Cancers in Europe (RARECARE)

## Abstract

Several classifications have been proposed to define rare cancers; however, the pathophysiological understanding of tumors evolves rapidly. We propose a New Classification of Rare Cancer (NCRC) using the updated International Classification of Diseases for Oncology 3.2 coding system and World Health Organization Classification of Tumors 5th edition. We applied patient data recorded in the National Cancer Registry of Japan to the new classification, setting a cut‐off of a crude incidence rate of 6 cases/100 000/year to define rare cancers, and developed a list of rare cancers in Japan from 2016 to 2019. The NCRC system identified various rare cancers, comprising 20.0% of all cancer diagnoses in this period. To examine this classification system's performance, we compared rare/non‐rare labeling of cancers by the Surveillance of Rare Cancers in Europe (RARECARENet) project and NCRC system. Compared with cases using the RARECARENet classification in Europe, 69 351 cases/year (6.8%) switched status with our classification, with 45 293 and 232 109 cases (4 years) switching from rare and non‐rare, respectively. Major differences included diffuse large B‐cell lymphomas, some thyroid cancers, oral cavity and lip cancers, and squamous cell carcinoma of the uterine cervix. As the NCRC includes newly classified tumor entities, it warrants validation using other diverse cohorts.

## Graphical abstract

We propose a new rare cancer classification system using the updated International Classification of Diseases for Oncology 3.2 coding system and the World Health Organization Classification of Tumors 5th edition. This list could serve as a diagnostic classifier to reliably distinguish various rare cancers for clinical applications. We applied patient data recorded in the National Cancer Registry of Japan to the current list, and showed the differences in rare/non‐rare classification from the Surveillance of Rare Cancers in Europe (RARECARENet) project.

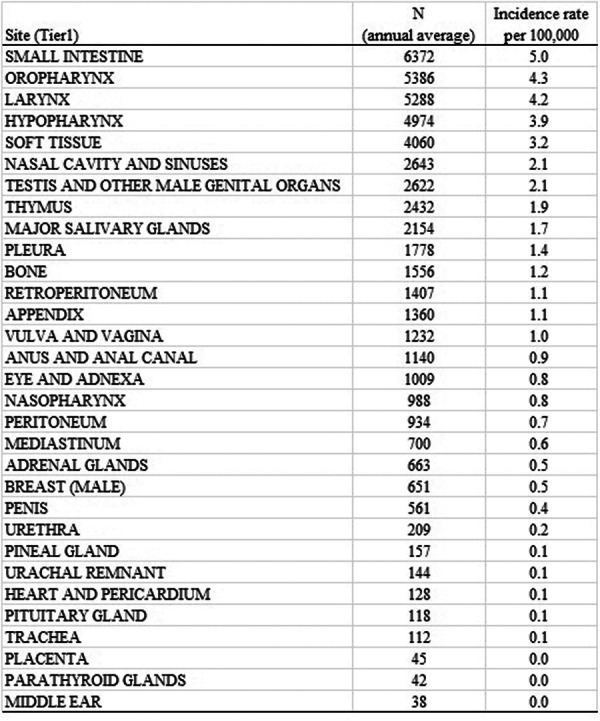

**Abbreviations**
ICD‐O3.2: International Classification of Diseases for Oncology 3.2
NCRC: new classification of rare cancers
RARECARENet: Surveillance of Rare Cancers in Europe

## 1 Introduction

Cancer comprises a heterogeneous array of diseases with varying manifestations depending on site and histology [1]. Despite advances in our understanding of cancer, rare cancers remain a challenge. Their rarity impedes research efforts and limits the development of diagnostic and treatment strategies. Therefore, systematic policy support is essential to address the disadvantages faced by patients with rare cancers.

A crucial first step in policy support is to clearly define what constitutes rare cancers. Several studies have attempted to define the corresponding frequency criteria. In the United States (US), rare cancers are defined as those with an annual incidence of < 15 cases/100 000/year [2] using Surveillance Epidemiology and End Results, with variables mainly based on the anatomical primary site supplemented by histological information [3]. In Europe, rare cancers are defined as those with an annual incidence of < 6 cases/100 000, according to the project Surveillance of Rare Cancers in Europe (RARECARENet) [4, 5]. The RARECARENet classification is more detailed than that used in the US, organized into three tumor classification tiers: Tier 1, by primary site and some site‐agnostic histological categories, including hematopoietic malignancies, soft tissue sarcomas, or neuroendocrine tumors; Tier 2, by histological groups relevant to clinical management and research; and Tier 3, which subdivides individual cancer entities with the corresponding International Classification of Diseases for Oncology 3 coding system (ICD‐O‐3) morphology, such as signet ring cell carcinoma [6]. In total, 198 rare cancers were identified according to the latest RARECARENet list [5].

Although the RARECARENet list provides a foundation for advancement in our classification of rare cancers, certain aspects can be improved. The list has not been updated since 2015 and does not incorporate the latest ICD‐O‐3.2 morphological codes, leading to the exclusion of newly identified tumors. Additionally, advances in biological and genomic research have altered disease concepts, providing the basis for some classifications. For instance, spindle cell morphology, previously associated with squamous cell carcinoma, is now considered closer to sarcoma [7]. Furthermore, some cancers are classified according to Tier 1 classification using histology, whereas Tier 2 groupings use anatomical sites to create inconsistencies.

This study proposes reconstructing the RARECARENet list into a new classification of rare cancers (NCRC). By incorporating the updates of the ICD‐O‐3.2 system and applying this revised classification to the Japanese National Cancer Registry (NCR) data, we aimed to identify rare cancers in Japan and evaluate differences in rare/non‐rare classifications resulting from the transition from the RARECARENet system to the NCRC system.

## 2 Materials and Methods

### 2.1 Development of the Classification

To develop a new classification system, we followed two main principles: (1) adopting a three‐tier system (Tier 1 to Tier 3), where Tier 1 is based strictly on the anatomical site of tumors; Tier 2, on a broad classification of histological groups within sites; and Tier 3, on more detailed histologies; and (2) the latest World Health Organization (WHO) classification of tumor terminology.

We used the ICD‐O‐3.2 released in 2019, consisting of topography and morphology codes. Topographic codes are four characters long (C00.0–C80.9), representing the site of the disease. The morphological codes are five digits (8000/0–9992/3) representing the disease histology. The fifth digit, after the slash (/), is the behavior code, which indicates either malignant invasive diseases (/3), in situ (/2), borderline malignancy (/1), or benign (/0). This classification focused mostly on malignant invasive diseases (/3) and several borderline diseases (/1). We excluded all benign tumors, in situ tumors, and, in principle, all borderline malignant tumors; however, we retained borderline tumors in the central nervous system (CNS) that are clinically aggressive and other borderline tumors that were recently changed from/3 to/1 in the revision from ICD‐O‐3.1 to 3.2.

In the three‐tier classification, Tier 1 uses only topographic codes; within each Tier 1 classification, we developed Tiers 2 and 3 based on the morphological codes of ICD‐O‐3.2. To create a consistent classification list across tumor sites, we developed two tables: one for site‐agnostic histology that can occur at any site (e.g., sarcoma and lymphoma) and another for site‐specific histology that occurs at selective sites (e.g., gastrointestinal stromal tumors). Some histologies are common to all sites but are treated differently depending on the site. Such histology appears in both tables (site‐specific and site‐agnostic), and the classification in the site‐specific table was applied to cases of the specified sites. Tier 2 within each Tier 1 classification was developed by combining site‐specific and site‐agnostic histologies. After drafting the classification, we tested it by applying it to cases diagnosed between 2016 and 2022 in the National Database of Hospital‐based Cancer Registries (HBCR) in Japan to identify any tumors that could not be captured in the new classifications. To determine the incidence of each tumor type, we applied the classification to the NCR data. We used the HBCR first because it started using ICD‐O‐3.2 in 2020, whereas the NCR still used ICD‐O‐3.1.

### 2.2 Data Sources

We used two cancer registries: HBCR and NCR. The HBCR was operated by “cancer care hospitals” designated by the Ministry of Health, Labour, and Welfare in Japan. The HBCR database covers approximately 70% of all newly diagnosed cancer patients in Japan and collects basic information, including date of birth, sex, tumor location, and pathology, using ICD‐O‐3.2 codes [8]. We used the NCR to calculate the incidence of individual cancers because the HBCR covers only patients from designated hospitals.

The NCR, established under the Cancer Registry Act in 2016, is a nationwide population‐based registry that collects basic information on all incident cancer cases in Japan. By law, all hospitals in Japan are mandated to report to the registry by the end of the year after the diagnosis. The NCR focuses on the comprehensive coverage of cancer cases with relatively simple information, such as ICD‐O‐3, date of diagnosis, and types of treatment received (e.g., surgery, chemotherapy, and radiation therapy). Data were made available for research use after formal review by the data utilization committee. Because the NCR used ICD‐O‐3.1, the data were converted to ICD‐O‐3.2 for the application of the NCRC. We posted an initial draft of the classifications on our website and invited comments from clinicians. We incorporated the comments raised during this process.

### 2.3 Data Analyses

We calculated the crude annual incidence rates by dividing the number of new cancer cases recorded in the NCR from January 1, 2016, to December 31, 2019, by the total Japanese population in the same period. Population data from 2016 to 2019 were obtained from the statistics produced by the Ministry of Internal Affairs and Communications in Japan [9]. When counting the incidences, we corrected specific cases with clear misclassifications or where metastatic sites were mistakenly registered as primary sites.

The number of cases in each Tier 1 group was calculated from the topographic codes, whereas that in Tiers 2 and 3 was based on morphologic codes. Some morphological codes, such as 8000 (malignant neoplasm, not otherwise specified (NOS)) or 8010 (carcinoma, NOS), do not specify the histology and were not assigned to any class in Tier 2. Consequently, cases with these morphologic codes were counted only in Tier 1; therefore, the number of cases in Tier 1 is not necessarily the sum of all Tier 2 groups.

We defined rare cancers as those with an incidence rate < 6/100 000/year at the Tier 1 and/or Tier 2 level. Owing to the hierarchical structure of Tiers 1 and 2, cases that are rare at Tier 1 are also rare at Tier 2; therefore, for a more detailed description, we defined cancers as “Tier 1‐Rare” when a tumor met the rarity criteria at the Tier 1 level, and “Tier 2‐Rare” when the tumor does not satisfy the rarity at Tier 1 level, but does at the Tier 2 level.

To compare the classification systems, we examined cases that had different rare/not‐rare labeling between the NCRC list and the RARECARENet list (downloaded from the RARECARENet homepage) using the RARECARENet classification revised in December 2015 [6]. All statistical analyses were performed using Stata MP/17 (College Station, TX, USA).

## 3 Results

### 3.1 NCRC and the List of Rare Cancers

The NCRC system developed in this study is presented in Supporting Information S1: Table S1 (A: morphologic code list and B: topographic code list). The NCRC list had 52 groupings in Tier 1 based on anatomical sites and 44 site‐agnostic and 59 site‐specific classifications in Tier 2 classifications based on pathohistological features.

**Figure 1:**
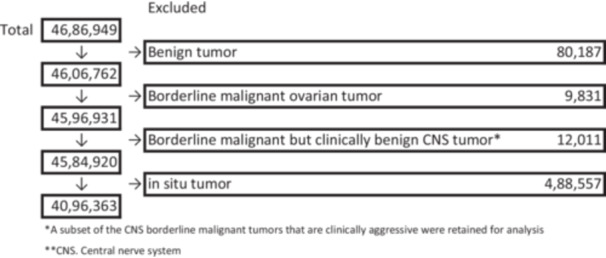
Schematic representation of study cases.

Figure 1 shows the process of selecting cases of tumors diagnosed between 2016 and 2019 and registered in the NCR databases. After excluding benign tumors and borderline malignant tumors, which are not included in the NCRC or RARECARE lists, 4 584 920 new malignant cases and borderline CNS tumors diagnosed between 2016 and 2019 (for 4 years) in Japan were identified. Several borderline CNS diseases (/1), which are not included in either the NCRC classification or RARECARE classification, were excluded, including 12 011 cases diagnosed between 2016 and 2019 in Japan (Figure 1); other CNS cases of “/1” were included in this analysis. We further excluded 488 557 cases of “in situ (/2)” diseases, leaving 4 096 363 cases for the analysis.

Tables 1 and 2 list “Tier 1‐Rare” and “Tier 2‐Rare” cancers, respectively. The number of rare cancer cases recorded in the NCR between 2016 and 2019 was 819 230. The total number of “Tier 1‐Rare” and “Tier 2‐Rare” cancer entities was 31 and 364, respectively. The proportion of all rare cancer entities was approximately 20.0% of all cancer diagnoses from 2016 to 2019. Table 3 presents a list of the common cancers; the number of common cancer cases recorded in the NCR from 2016 to 2019 was 3 229 363 cases. The total number of common cancer entities was 19, corresponding to approximately 78.8% of all cancer diagnoses.

**Table 1:** List of Tier 1 rare cancers.

Site (Tier 1)	*N* (annual average)	Incidence rate per 100 000
SMALL INTESTINE	6372	5.0
OROPHARYNX	5386	4.3
LARYNX	5288	4.2
HYPOPHARYNX	4974	3.9
SOFT TISSUE	4060	3.2
NASAL CAVITY AND SINUSES	2643	2.1
TESTIS AND OTHER MALE GENITAL ORGANS	2622	2.1
THYMUS	2432	1.9
MAJOR SALIVARY GLANDS	2154	1.7
PLEURA	1778	1.4
BONE	1556	1.2
RETROPERITONEUM	1407	1.1
APPENDIX	1360	1.1
VULVA AND VAGINA	1232	1.0
ANUS AND ANAL CANAL	1140	0.9
EYE AND ADNEXA	1009	0.8
NASOPHARYNX	988	0.8
PERITONEUM	934	0.7
MEDIASTINUM	700	0.6
ADRENAL GLANDS	663	0.5
BREAST (MALE)	651	0.5
PENIS	561	0.4
URETHRA	209	0.2
PINEAL GLAND	157	0.1
URACHAL REMNANT	144	0.1
HEART AND PERICARDIUM	128	0.1
PITUITARY GLAND	118	0.1
TRACHEA	112	0.1
PLACENTA	45	0.0
PARATHYROID GLANDS	42	0.0
MIDDLE EAR	38	0.0

**Table 2:** List of Tier 2 rare cancers.

Site (Tier 1)	Histology (Tier 2)	*N* (annual average)	Incidence rate per 100 000
STOMACH	GIST	3868	3.1
STOMACH	Lymphoid diseases	2826	2.2
STOMACH	Neuroendocrine carcinoma, NOS	530	0.4
STOMACH	Neuroendocrine tumor, NOS	466	0.4
STOMACH	Carcinoma, special type	344	0.3
STOMACH	Undifferentiated carcinoma, NOS	138	0.1
STOMACH	Mixed neuroendocrine non‐neuroendocrine neoplasm	95	< 0.1
STOMACH	Lymphoepithelioma(‐like) carcinoma	87	< 0.1
STOMACH	Sarcoma	49	< 0.1
STOMACH	Germ cell associated tumor	< 10	< 0.1
STOMACH	Mucosa and extracutaneous melanoma	< 10	< 0.1
STOMACH	Carcinosarcoma/Sarcomatoid carcinoma, NOS	< 10	< 0.1
STOMACH	Salivary gland type carcinoma	< 10	< 0.1
STOMACH	Histiocytic and dendritic cell neoplasms	< 10	< 0.1
STOMACH	Acute myeloid leukaemia and related precursor neoplasms	< 10	< 0.1
LUNG	(Non‐small cell carcinoma, NOS)	3076	2.4
LUNG	Adenocarcinoma, special type	2171	1.7
LUNG	Adenosquamous carcinoma	870	0.7
LUNG	Carcinosarcoma/Sarcomatoid carcinoma, NOS	714	0.6
LUNG	Lymphoid diseases	533	0.4
LUNG	Large cell carcinoma	462	0.4
LUNG	Mixed neuroendocrine non‐neuroendocrine neoplasm	384	0.3
LUNG	Neuroendocrine tumor, NOS	382	0.3
LUNG	Salivary gland type carcinoma	193	0.2
LUNG	Sarcoma	81	< 0.1
LUNG	Undifferentiated carcinoma, NOS	60	< 0.1
LUNG	Mucosa and extracutaneous melanoma	11	< 0.1
LUNG	Histiocytic and dendritic cell neoplasms	11	< 0.1
LUNG	Lymphoepithelioma(‐like) carcinoma	11	< 0.1
LUNG	Pulmonary blastoma	< 10	< 0.1
LUNG	Germ cell associated tumor	< 10	< 0.1
LUNG	Pleuropulmonary blastoma	< 10	< 0.1
LUNG	Paraganglioma, NOS, malignant	< 10	< 0.1
LUNG	Neuroblastoma	< 10	< 0.1
COLON	Adenocarcinoma, special type	3433	2.7
COLON	Lymphoid diseases	612	0.5
COLON	Neuroendocrine carcinoma, NOS	177	0.1
COLON	Neuroendocrine tumor, NOS	143	0.1
COLON	Mixed neuroendocrine non‐neuroendocrine neoplasm	44	< 0.1
COLON	GIST	43	< 0.1
COLON	Sarcoma	32	< 0.1
COLON	Undifferentiated carcinoma, NOS	32	< 0.1
COLON	Squamous cell carcinoma	30	< 0.1
COLON	Adenocarcinoid tumor	< 10	< 0.1
COLON	Salivary gland type carcinoma	< 10	< 0.1
COLON	Mucosa and extracutaneous melanoma	< 10	< 0.1
COLON	Carcinosarcoma/Sarcomatoid carcinoma, NOS	< 10	< 0.1
COLON	Germ cell associated tumor	< 10	< 0.1
COLON	Lymphoepithelioma(‐like) carcinoma	< 10	< 0.1
COLON	Histiocytic and dendritic cell neoplasms	< 10	< 0.1
BREAST (FEMALE)	Special types of adenocarcinoma of female breast	5742	4.5
BREAST (FEMALE)	Invasive lobular carcinoma of female breast	4675	3.7
BREAST (FEMALE)	Metaplastic carcinoma of female breast	416	0.3
BREAST (FEMALE)	Lymphoid diseases	302	0.2
BREAST (FEMALE)	Phyllodes tumor, malignant, of female breast	183	0.1
BREAST (FEMALE)	Mammary Paget's disease of female breast	142	0.1
BREAST (FEMALE)	Salivary gland type carcinoma	79	< 0.1
BREAST (FEMALE)	Sarcoma	52	< 0.1
BREAST (FEMALE)	Neuroendocrine carcinoma, NOS	< 10	< 0.1
BREAST (FEMALE)	Carcinosarcoma/Sarcomatoid carcinoma, NOS	< 10	< 0.1
BREAST (FEMALE)	Undifferentiated carcinoma, NOS	< 10	< 0.1
BREAST (FEMALE)	Adenomyoepithelioma with carcinoma of female breast	< 10	< 0.1
BREAST (FEMALE)	Mucosa and extracutaneous melanoma	< 10	< 0.1
BREAST (FEMALE)	Lymphoepithelioma(‐like) carcinoma	< 10	< 0.1
BREAST (FEMALE)	Acute myeloid leukaemia and related precursor neoplasms	< 10	< 0.1
BREAST (FEMALE)	Neuroendocrine tumor, NOS	< 10	< 0.1
PROSTATE GLAND	Prostatic ductal carcinoma	193	0.2
PROSTATE GLAND	Neuroendocrine carcinoma, NOS	114	< 0.1
PROSTATE GLAND	Squamous cell carcinoma	44	< 0.1
PROSTATE GLAND	Undifferentiated carcinoma, NOS	38	< 0.1
PROSTATE GLAND	Lymphoid diseases	36	< 0.1
PROSTATE GLAND	Sarcoma	20	< 0.1
PROSTATE GLAND	Mixed neuroendocrine non‐neuroendocrine neoplasm	< 10	< 0.1
PROSTATE GLAND	Basal cell carcinoma	< 10	< 0.1
PROSTATE GLAND	Stromal Sarcoma	< 10	< 0.1
PROSTATE GLAND	Carcinosarcoma/Sarcomatoid carcinoma, NOS	< 10	< 0.1
PROSTATE GLAND	Germ cell associated tumor	< 10	< 0.1
PROSTATE GLAND	Neuroendocrine tumor, NOS	< 10	< 0.1
PROSTATE GLAND	Mucosa and extracutaneous melanoma	< 10	< 0.1
PANCREAS	Neuroendocrine tumor, NOS	1388	1.1
PANCREAS	Adenocarcinoma, special type	1281	1.0
PANCREAS	Neuroendocrine carcinoma, NOS	212	0.2
PANCREAS	Pancreatic anaplastic carcinoma	177	0.1
PANCREAS	Lymphoid diseases	109	< 0.1
PANCREAS	Squamous cell carcinoma	49	< 0.1
PANCREAS	Mixed neuroendocrine non‐neuroendocrine neoplasm	28	< 0.1
PANCREAS	Sarcoma	13	< 0.1
PANCREAS	GIST	< 10	< 0.1
PANCREAS	Pancreatoblastoma	< 10	< 0.1
PANCREAS	Carcinosarcoma/Sarcomatoid carcinoma, NOS	< 10	< 0.1
PANCREAS	Mucosa and extracutaneous melanoma	< 10	< 0.1
PANCREAS	Paraganglioma, NOS, malignant	< 10	< 0.1
PANCREAS	Salivary gland type carcinoma	< 10	< 0.1
PANCREAS	Germ cell associated tumor	< 10	< 0.1
PANCREAS	Histiocytic and dendritic cell neoplasms	< 10	< 0.1
PANCREAS	Neuroblastoma	< 10	< 0.1
LIVER AND INTRAHEPATIC BILE TRACT	Adenocarcinoma, usual type	5612	4.4
LIVER AND INTRAHEPATIC BILE TRACT	Lymphoid diseases	203	0.2
LIVER AND INTRAHEPATIC BILE TRACT	Sarcoma	88	< 0.1
LIVER AND INTRAHEPATIC BILE TRACT	Adenocarcinoma, special type	73	< 0.1
LIVER AND INTRAHEPATIC BILE TRACT	Hepatoblastoma	53	< 0.1
LIVER AND INTRAHEPATIC BILE TRACT	Neuroendocrine carcinoma, NOS	48	< 0.1
LIVER AND INTRAHEPATIC BILE TRACT	Neuroendocrine tumor, NOS	28	< 0.1
LIVER AND INTRAHEPATIC BILE TRACT	Squamous cell carcinoma	< 10	< 0.1
LIVER AND INTRAHEPATIC BILE TRACT	Undifferentiated embryonal sarcoma	< 10	< 0.1
LIVER AND INTRAHEPATIC BILE TRACT	Carcinosarcoma/Sarcomatoid carcinoma, NOS	< 10	< 0.1
LIVER AND INTRAHEPATIC BILE TRACT	Undifferentiated carcinoma, NOS	< 10	< 0.1
LIVER AND INTRAHEPATIC BILE TRACT	Hepatocellular carcinoma, fibrolamellar	< 10	< 0.1
LIVER AND INTRAHEPATIC BILE TRACT	Lymphoepithelioma(‐like) carcinoma	< 10	< 0.1
LIVER AND INTRAHEPATIC BILE TRACT	Mucosa and extracutaneous melanoma	< 10	< 0.1
LIVER AND INTRAHEPATIC BILE TRACT	Mixed neuroendocrine non‐neuroendocrine neoplasm	< 10	< 0.1
LIVER AND INTRAHEPATIC BILE TRACT	Histiocytic and dendritic cell neoplasms	< 10	< 0.1
LIVER AND INTRAHEPATIC BILE TRACT	Germ cell associated tumor	< 10	< 0.1
LIVER AND INTRAHEPATIC BILE TRACT	Salivary gland type carcinoma	< 10	< 0.1
LIVER AND INTRAHEPATIC BILE TRACT	GIST	< 10	< 0.1
LIVER AND INTRAHEPATIC BILE TRACT	Neuroblastoma	< 10	< 0.1
LIVER AND INTRAHEPATIC BILE TRACT	Paraganglioma, NOS, malignant	< 10	< 0.1
RECTUM	Neuroendocrine tumor, NOS	3052	2.4
RECTUM	Adenocarcinoma, special type	714	0.6
RECTUM	Lymphoid diseases	209	0.2
RECTUM	GIST	186	0.1
RECTUM	Neuroendocrine carcinoma, NOS	150	0.1
RECTUM	Mucosa and extracutaneous melanoma	59	< 0.1
RECTUM	Squamous cell carcinoma	53	< 0.1
RECTUM	Mixed neuroendocrine non‐neuroendocrine neoplasm	20	< 0.1
RECTUM	Sarcoma	13	< 0.1
RECTUM	Undifferentiated carcinoma, NOS	< 10	< 0.1
RECTUM	Adenocarcinoid tumor	< 10	< 0.1
RECTUM	Salivary gland type carcinoma	< 10	< 0.1
RECTUM	Germ cell associated tumor	< 10	< 0.1
HEMATOPOIETIC AND RETICULOENDOTHELIAL SYSTEMS	Plasmacytoma/Multiple Myeloma (and Heavy chain diseases)	7133	5.6
HEMATOPOIETIC AND RETICULOENDOTHELIAL SYSTEMS	Myelodysplastic syndrome, unclassifiable	6287	5.0
HEMATOPOIETIC AND RETICULOENDOTHELIAL SYSTEMS	Acute myeloid leukaemia	6105	4.8
HEMATOPOIETIC AND RETICULOENDOTHELIAL SYSTEMS	Other myeloproliferative neoplasms	4256	3.4
HEMATOPOIETIC AND RETICULOENDOTHELIAL SYSTEMS	Myelodysplastic syndrome with ring sideroblasts	2628	2.1
HEMATOPOIETIC AND RETICULOENDOTHELIAL SYSTEMS	Chronic myeloid leukaemia	2526	2.0
HEMATOPOIETIC AND RETICULOENDOTHELIAL SYSTEMS	Precursor B/T lymphoblastic leukaemia/lymphoblastic lymphoma (and Burkitt leukaemia/lymphoma)	1828	1.4
HEMATOPOIETIC AND RETICULOENDOTHELIAL SYSTEMS	Other non‐Hodgkin mature B‐cell lymphoma	1426	1.1
HEMATOPOIETIC AND RETICULOENDOTHELIAL SYSTEMS	Adult T‐cell leukaemia/lymphoma	1069	0.8
HEMATOPOIETIC AND RETICULOENDOTHELIAL SYSTEMS	Myelodysplastic syndrome with single lineage dysplasia	810	0.6
HEMATOPOIETIC AND RETICULOENDOTHELIAL SYSTEMS	Lymphoid diseases	557	0.4
HEMATOPOIETIC AND RETICULOENDOTHELIAL SYSTEMS	Chronic Myelomonocytic leukaemia	437	0.3
HEMATOPOIETIC AND RETICULOENDOTHELIAL SYSTEMS	Acute leukaemia, NOS	312	0.2
HEMATOPOIETIC AND RETICULOENDOTHELIAL SYSTEMS	Myeloid proliferations associated with Down syndrome	233	0.2
HEMATOPOIETIC AND RETICULOENDOTHELIAL SYSTEMS	Other T cell lymphomas and NK cell neoplasms	157	0.1
HEMATOPOIETIC AND RETICULOENDOTHELIAL SYSTEMS	Diffuse large B‐cell lymphoma	132	0.1
HEMATOPOIETIC AND RETICULOENDOTHELIAL SYSTEMS	Acute leukaemia, mixed phenotype	86	< 0.1
HEMATOPOIETIC AND RETICULOENDOTHELIAL SYSTEMS	Hairy cell leukaemia	63	< 0.1
HEMATOPOIETIC AND RETICULOENDOTHELIAL SYSTEMS	Non‐Hodgkin lymphoma, NOS	61	< 0.1
HEMATOPOIETIC AND RETICULOENDOTHELIAL SYSTEMS	Prolymphocytic leukaemia	59	< 0.1
HEMATOPOIETIC AND RETICULOENDOTHELIAL SYSTEMS	Histiocytic and dendritic cell neoplasms	24	< 0.1
HEMATOPOIETIC AND RETICULOENDOTHELIAL SYSTEMS	Follicular lymphoma	20	< 0.1
HEMATOPOIETIC AND RETICULOENDOTHELIAL SYSTEMS	Mantle cell lymphoma	18	< 0.1
HEMATOPOIETIC AND RETICULOENDOTHELIAL SYSTEMS	Malignant lymphoma, NOS	17	< 0.1
HEMATOPOIETIC AND RETICULOENDOTHELIAL SYSTEMS	Hodgkin lymphoma, classical	< 10	< 0.1
HEMATOPOIETIC AND RETICULOENDOTHELIAL SYSTEMS	Myeloid/lymphoid neoplasms with eosinophilia and gene rearrangement	< 10	< 0.1
HEMATOPOIETIC AND RETICULOENDOTHELIAL SYSTEMS	Mast cell tumor	< 10	< 0.1
HEMATOPOIETIC AND RETICULOENDOTHELIAL SYSTEMS	Anaplastic large cell lymphoma	< 10	< 0.1
HEMATOPOIETIC AND RETICULOENDOTHELIAL SYSTEMS	Hodgkin lymphoma nodular lymphocyte predominance	< 10	< 0.1
HEMATOPOIETIC AND RETICULOENDOTHELIAL SYSTEMS	Lymphoproliferative disease, NOS	< 10	< 0.1
HEMATOPOIETIC AND RETICULOENDOTHELIAL SYSTEMS	Cutaneous T cell lymphoma (Sezary syn, Mycosis fung)	< 10	< 0.1
HEMATOPOIETIC AND RETICULOENDOTHELIAL SYSTEMS	Sarcoma	< 10	< 0.1
SKIN AND ADNEXAL	Malignant skin melanoma	1828	1.4
SKIN AND ADNEXAL	Adnexal carcinoma	1455	1.1
SKIN AND ADNEXAL	Lymphoid diseases	1340	1.1
SKIN AND ADNEXAL	Sarcoma	586	0.5
SKIN AND ADNEXAL	Merkel cell carcinoma	236	0.2
SKIN AND ADNEXAL	Salivary gland type carcinoma	61	< 0.1
SKIN AND ADNEXAL	Histiocytic and dendritic cell neoplasms	27	< 0.1
SKIN AND ADNEXAL	Neuroendocrine carcinoma, NOS	< 10	< 0.1
SKIN AND ADNEXAL	Lymphoepithelioma(‐like) carcinoma	< 10	< 0.1
SKIN AND ADNEXAL	Undifferentiated carcinoma, NOS	< 10	< 0.1
SKIN AND ADNEXAL	Carcinosarcoma/Sarcomatoid carcinoma, NOS	< 10	< 0.1
SKIN AND ADNEXAL	Acute myeloid leukaemia and related precursor neoplasms	< 10	< 0.1
SKIN AND ADNEXAL	Neuroendocrine tumor, NOS	< 10	< 0.1
SKIN AND ADNEXAL	Germ cell associated tumor	< 10	< 0.1
OESOPHAGUS	Adenocarcinoma, usual type	1868	1.5
OESOPHAGUS	Neuroendocrine carcinoma, NOS	237	0.2
OESOPHAGUS	Carcinosarcoma/Sarcomatoid carcinoma, NOS	68	< 0.1
OESOPHAGUS	Mucosa and extracutaneous melanoma	59	< 0.1
OESOPHAGUS	GIST	49	< 0.1
OESOPHAGUS	Lymphoid diseases	23	< 0.1
OESOPHAGUS	Undifferentiated carcinoma, NOS	21	< 0.1
OESOPHAGUS	Mixed neuroendocrine non‐neuroendocrine neoplasm	14	< 0.1
OESOPHAGUS	Sarcoma	13	< 0.1
OESOPHAGUS	Salivary gland type carcinoma	< 10	< 0.1
OESOPHAGUS	Neuroendocrine tumor, NOS	< 10	< 0.1
OESOPHAGUS	Lymphoepithelioma(‐like) carcinoma	< 10	< 0.1
OESOPHAGUS	Germ cell associated tumor	< 10	< 0.1
OESOPHAGUS	Acute myeloid leukaemia and related precursor neoplasms	< 10	< 0.1
OESOPHAGUS	Histiocytic and dendritic cell neoplasms	< 10	< 0.1
BLADDER	Adenocarcinoma	283	0.2
BLADDER	Squamous cell carcinoma	253	0.2
BLADDER	Neuroendocrine carcinoma, NOS	189	0.1
BLADDER	Lymphoid diseases	74	< 0.1
BLADDER	Sarcoma	24	< 0.1
BLADDER	Undifferentiated carcinoma, NOS	22	< 0.1
BLADDER	Carcinosarcoma/Sarcomatoid carcinoma, NOS	14	< 0.1
BLADDER	Lymphoepithelioma(‐like) carcinoma	11	< 0.1
BLADDER	Paraganglioma, NOS, malignant	< 10	< 0.1
BLADDER	Neuroendocrine tumor, NOS	< 10	< 0.1
BLADDER	Mixed neuroendocrine non‐neuroendocrine neoplasm	< 10	< 0.1
BLADDER	Neuroblastoma	< 10	< 0.1
BLADDER	Germ cell associated tumor	< 10	< 0.1
BLADDER	GIST	< 10	< 0.1
BLADDER	Salivary gland type carcinoma	< 10	< 0.1
BLADDER	Mucosa and extracutaneous melanoma	< 10	< 0.1
GALLBLADDER and EXTRAHEPATIC BILIARY TRACT	Neuroendocrine carcinoma, NOS	135	0.1
GALLBLADDER and EXTRAHEPATIC BILIARY TRACT	Squamous cell carcinoma	66	< 0.1
GALLBLADDER and EXTRAHEPATIC BILIARY TRACT	Neuroendocrine tumor, NOS	60	< 0.1
GALLBLADDER and EXTRAHEPATIC BILIARY TRACT	Adenocarcinoma, special type	49	< 0.1
GALLBLADDER and EXTRAHEPATIC BILIARY TRACT	Undifferentiated carcinoma, NOS	38	< 0.1
GALLBLADDER and EXTRAHEPATIC BILIARY TRACT	Mixed neuroendocrine non‐neuroendocrine neoplasm	33	< 0.1
GALLBLADDER and EXTRAHEPATIC BILIARY TRACT	Lymphoid diseases	30	< 0.1
GALLBLADDER and EXTRAHEPATIC BILIARY TRACT	Carcinosarcoma/Sarcomatoid carcinoma, NOS	24	< 0.1
GALLBLADDER and EXTRAHEPATIC BILIARY TRACT	Sarcoma	< 10	< 0.1
GALLBLADDER and EXTRAHEPATIC BILIARY TRACT	Mucosa and extracutaneous melanoma	< 10	< 0.1
GALLBLADDER and EXTRAHEPATIC BILIARY TRACT	Germ cell associated tumor	< 10	< 0.1
GALLBLADDER and EXTRAHEPATIC BILIARY TRACT	Salivary gland type carcinoma	< 10	< 0.1
GALLBLADDER and EXTRAHEPATIC BILIARY TRACT	Histiocytic and dendritic cell neoplasms	< 10	< 0.1
GALLBLADDER and EXTRAHEPATIC BILIARY TRACT	GIST	< 10	< 0.1
GALLBLADDER and EXTRAHEPATIC BILIARY TRACT	Paraganglioma, NOS, malignant	< 10	< 0.1
KIDNEY	Renal cell carcinoma, special type	1903	1.5
KIDNEY	Lymphoid diseases	89	< 0.1
KIDNEY	Collecting duct carcinoma	67	< 0.1
KIDNEY	Sarcoma	53	< 0.1
KIDNEY	Nephroblastoma	48	< 0.1
KIDNEY	Neuroendocrine tumor, NOS	< 10	< 0.1
KIDNEY	Neuroendocrine carcinoma, NOS	< 10	< 0.1
KIDNEY	Undifferentiated carcinoma, NOS	< 10	< 0.1
KIDNEY	Squamous cell carcinoma, NOS	< 10	< 0.1
KIDNEY	Rhabdoid tumor, NOS	< 10	< 0.1
KIDNEY	Neuroblastoma	< 10	< 0.1
KIDNEY	Mucinous tubular and spindle cell carcinoma	< 10	< 0.1
KIDNEY	Mucosa and extracutaneous melanoma	< 10	< 0.1
KIDNEY	Germ cell associated tumor	< 10	< 0.1
KIDNEY	Carcinosarcoma/Sarcomatoid carcinoma, NOS	< 10	< 0.1
KIDNEY	Salivary gland type carcinoma	< 10	< 0.1
LYMPH NODE	Diffuse large B‐cell lymphoma	7068	5.6
LYMPH NODE	Follicular lymphoma	4124	3.3
LYMPH NODE	Malignant lymphoma, NOS	2672	2.1
LYMPH NODE	Other T cell lymphomas and NK cell neoplasms	1456	1.2
LYMPH NODE	Hodgkin lymphoma, classical	1164	0.9
LYMPH NODE	Other non‐Hodgkin mature B‐cell lymphoma	936	0.7
LYMPH NODE	Non‐Hodgkin lymphoma, NOS	794	0.6
LYMPH NODE	Mantle cell lymphoma	465	0.4
LYMPH NODE	Precursor B/T lymphoblastic leukaemia/lymphoblastic lymphoma (and Burkitt leukaemia/lymphoma)	193	0.2
LYMPH NODE	Hodgkin lymphoma nodular lymphocyte predominance	169	0.1
LYMPH NODE	Adult T‐cell leukaemia/lymphoma	154	0.1
LYMPH NODE	Anaplastic large cell lymphoma	127	0.1
LYMPH NODE	Sarcoma	15	< 0.1
LYMPH NODE	Histiocytic and dendritic cell neoplasms	12	< 0.1
LYMPH NODE	Plasmacytoma/Multiple Myeloma (and Heavy chain diseases)	< 10	< 0.1
LYMPH NODE	Cutaneous T cell lymphoma (Sezary syn, Mycosis fung)	< 10	< 0.1
LYMPH NODE	Lymphoproliferative disease, NOS	< 10	< 0.1
LYMPH NODE	Acute myeloid leukaemia	< 10	< 0.1
THYROID GLAND	Lymphoid diseases	527	0.4
THYROID GLAND	Undifferentiated/anaplastic carcinoma	326	0.3
THYROID GLAND	Medullary thyroid carcinoma	216	0.2
THYROID GLAND	Thyroid carcinoma, special type	95	< 0.1
THYROID GLAND	Intrathyroid thymic carcinoma	< 10	< 0.1
THYROID GLAND	Sarcoma	< 10	< 0.1
THYROID GLAND	Salivary gland type carcinoma	< 10	< 0.1
THYROID GLAND	Neuroendocrine tumor, NOS	< 10	< 0.1
THYROID GLAND	Neuroendocrine carcinoma, NOS	< 10	< 0.1
THYROID GLAND	Histiocytic and dendritic cell neoplasms	< 10	< 0.1
CORPUS UTERI	Adenocarcinoma, special type	1589	1.3
CORPUS UTERI	Sarcoma	487	0.4
CORPUS UTERI	Clear cell adenocarcinoma, NOS	345	0.3
CORPUS UTERI	Endometrial stromal tumor	276	0.2
CORPUS UTERI	Undifferentiated carcinoma, NOS	92	< 0.1
CORPUS UTERI	Adenosarcoma	77	< 0.1
CORPUS UTERI	Neuroendocrine carcinoma, NOS	55	< 0.1
CORPUS UTERI	Squamous cell carcinoma	52	< 0.1
CORPUS UTERI	Lymphoid diseases	32	< 0.1
CORPUS UTERI	Germ cell associated tumor	< 10	< 0.1
CORPUS UTERI	Neuroendocrine tumor, NOS	< 10	< 0.1
CORPUS UTERI	Mixed neuroendocrine non‐neuroendocrine neoplasm	< 10	< 0.1
CORPUS UTERI	Lymphoepithelioma(‐like) carcinoma	< 10	< 0.1
CORPUS UTERI	Mucosa and extracutaneous melanoma	< 10	< 0.1
CORPUS UTERI	Salivary gland type carcinoma	< 10	< 0.1
OVARY AND OTHER FEMALE GENITAL ORGANS	Adenocarcinoma, usual type	5647	4.5
OVARY AND OTHER FEMALE GENITAL ORGANS	Clear cell carcinoma of ovary	2005	1.6
OVARY AND OTHER FEMALE GENITAL ORGANS	Adenocarcinoma, special type	1155	0.9
OVARY AND OTHER FEMALE GENITAL ORGANS	Germ cell associated tumor	378	0.3
OVARY AND OTHER FEMALE GENITAL ORGANS	Carcinosarcoma/Sarcomatoid carcinoma, NOS	158	0.1
OVARY AND OTHER FEMALE GENITAL ORGANS	Sex cord‐stromal tumors	107	< 0.1
OVARY AND OTHER FEMALE GENITAL ORGANS	Neuroendocrine tumor, NOS	39	< 0.1
OVARY AND OTHER FEMALE GENITAL ORGANS	Undifferentiated carcinoma, NOS	37	< 0.1
OVARY AND OTHER FEMALE GENITAL ORGANS	Sarcoma	24	< 0.1
OVARY AND OTHER FEMALE GENITAL ORGANS	Lymphoid diseases	19	< 0.1
OVARY AND OTHER FEMALE GENITAL ORGANS	Ovarian Small cell carcinoma with hypercalcemic type	15	< 0.1
OVARY AND OTHER FEMALE GENITAL ORGANS	Neuroendocrine carcinoma, NOS	11	< 0.1
OVARY AND OTHER FEMALE GENITAL ORGANS	Adenosarcoma	< 10	< 0.1
OVARY AND OTHER FEMALE GENITAL ORGANS	Mucosa and extracutaneous melanoma	< 10	< 0.1
OVARY AND OTHER FEMALE GENITAL ORGANS	Mixed neuroendocrine non‐neuroendocrine neoplasm	< 10	< 0.1
OVARY AND OTHER FEMALE GENITAL ORGANS	Salivary gland type carcinoma	< 10	< 0.1
OVARY AND OTHER FEMALE GENITAL ORGANS	Acute myeloid leukaemia and related precursor neoplasms	< 10	< 0.1
OVARY AND OTHER FEMALE GENITAL ORGANS	Lymphoepithelioma(‐like) carcinoma	< 10	< 0.1
OVARY AND OTHER FEMALE GENITAL ORGANS	Neuroblastoma	< 10	< 0.1
CERVIX UTERI	Squamous cell carcinoma	7578	6.0
CERVIX UTERI	Adenocarcinoma, usual type	1977	1.6
CERVIX UTERI	Adenocarcinoma, special type	687	0.5
CERVIX UTERI	Neuroendocrine carcinoma, NOS	136	0.1
CERVIX UTERI	Lymphoid diseases	36	< 0.1
CERVIX UTERI	Carcinosarcoma/Sarcomatoid carcinoma, NOS	27	< 0.1
CERVIX UTERI	Undifferentiated carcinoma, NOS	16	< 0.1
CERVIX UTERI	Sarcoma	15	< 0.1
CERVIX UTERI	Lymphoepithelioma(‐like) carcinoma	12	< 0.1
CERVIX UTERI	Mucosa and extracutaneous melanoma	< 10	< 0.1
CERVIX UTERI	Mixed neuroendocrine non‐neuroendocrine neoplasm	< 10	< 0.1
CERVIX UTERI	Adenosarcoma	< 10	< 0.1
CERVIX UTERI	Salivary gland type carcinoma	< 10	< 0.1
CERVIX UTERI	Neuroendocrine tumor, NOS	< 10	< 0.1
CERVIX UTERI	Acute myeloid leukaemia and related precursor neoplasms	< 10	< 0.1
CERVIX UTERI	Germ cell associated tumor	< 10	< 0.1
CERVIX UTERI	Histiocytic and dendritic cell neoplasms	< 10	< 0.1
ORAL CAVITY AND LIP	Salivary gland type carcinoma	302	0.2
ORAL CAVITY AND LIP	Lymphoid diseases	206	0.2
ORAL CAVITY AND LIP	Mucosa and extracutaneous melanoma	54	< 0.1
ORAL CAVITY AND LIP	Adenocarcinoma	45	< 0.1
ORAL CAVITY AND LIP	Sarcoma	33	< 0.1
ORAL CAVITY AND LIP	Odontogenic tumor	32	< 0.1
ORAL CAVITY AND LIP	Undifferentiated carcinoma, NOS	< 10	< 0.1
ORAL CAVITY AND LIP	Neuroendocrine carcinoma, NOS	< 10	< 0.1
ORAL CAVITY AND LIP	Carcinosarcoma/Sarcomatoid carcinoma, NOS	< 10	< 0.1
ORAL CAVITY AND LIP	Lymphoepithelioma(‐like) carcinoma	< 10	< 0.1
ORAL CAVITY AND LIP	Acute myeloid leukaemia and related precursor neoplasms	< 10	< 0.1
ORAL CAVITY AND LIP	Neuroendocrine tumor, NOS	< 10	< 0.1
ORAL CAVITY AND LIP	Histiocytic and dendritic cell neoplasms	< 10	< 0.1
RENAL PELVIS AND URETER	Urothelial carcinoma	6697	5.3
RENAL PELVIS AND URETER	Squamous cell carcinoma	101	< 0.1
RENAL PELVIS AND URETER	Adenocarcinoma	54	< 0.1
RENAL PELVIS AND URETER	Neuroendocrine carcinoma, NOS	37	< 0.1
RENAL PELVIS AND URETER	Lymphoid diseases	24	< 0.1
RENAL PELVIS AND URETER	Carcinosarcoma/Sarcomatoid carcinoma, NOS	< 10	< 0.1
RENAL PELVIS AND URETER	Undifferentiated carcinoma, NOS	< 10	< 0.1
RENAL PELVIS AND URETER	Sarcoma	< 10	< 0.1
RENAL PELVIS AND URETER	Lymphoepithelioma(‐like) carcinoma	< 10	< 0.1
RENAL PELVIS AND URETER	Neuroendocrine tumor, NOS	< 10	< 0.1
RENAL PELVIS AND URETER	Neuroblastoma	< 10	< 0.1
RENAL PELVIS AND URETER	Mucosa and extracutaneous melanoma	< 10	< 0.1
CENTRAL NERVOUS SYSTEM	Astrocytic tumors	3052	2.4
CENTRAL NERVOUS SYSTEM	Lymphoid diseases	1176	0.9
CENTRAL NERVOUS SYSTEM	Glioma, NOS	654	0.5
CENTRAL NERVOUS SYSTEM	Meningiomas, NOS	563	0.4
CENTRAL NERVOUS SYSTEM	Oligodendroglial tumors	336	0.3
CENTRAL NERVOUS SYSTEM	Hemangioblastoma	335	0.3
CENTRAL NERVOUS SYSTEM	Ependymal tumors	271	0.2
CENTRAL NERVOUS SYSTEM	Sarcoma	162	0.1
CENTRAL NERVOUS SYSTEM	Neuronal and mixed neuronal‐glial tumors	101	< 0.1
CENTRAL NERVOUS SYSTEM	Medulloblastoma	91	< 0.1
CENTRAL NERVOUS SYSTEM	Germ cell associated tumor	86	< 0.1
CENTRAL NERVOUS SYSTEM	Circumscribed astrocytic gliomas	61	< 0.1
CENTRAL NERVOUS SYSTEM	Craniopharyngioma, NOS	56	< 0.1
CENTRAL NERVOUS SYSTEM	Other CNS embryonal tumors	33	< 0.1
CENTRAL NERVOUS SYSTEM	Mucosa and extracutaneous melanoma	< 10	< 0.1
CENTRAL NERVOUS SYSTEM	Paraganglioma, NOS, malignant	< 10	< 0.1
CENTRAL NERVOUS SYSTEM	Choroid plexus carcinoma of CNS	< 10	< 0.1
CENTRAL NERVOUS SYSTEM	Histiocytic and dendritic cell neoplasms	< 10	< 0.1
CENTRAL NERVOUS SYSTEM	Neuroblastoma	< 10	< 0.1
CENTRAL NERVOUS SYSTEM	Paediatric‐type diffuse low‐grade gliomas	< 10	< 0.1
CENTRAL NERVOUS SYSTEM	Acute myeloid leukaemia and related precursor neoplasms	< 10	< 0.1
CENTRAL NERVOUS SYSTEM	Salivary gland type carcinoma	< 10	< 0.1

**Table 3:** List of common cancers.

Site (Tier 1)	Histology (Tier 2)	*N* (annual average)	Incidence rate per 100 000
STOMACH	Adenocarcinoma, usual type	118 733	93.8
COLON	Adenocarcinoma, usual type	103 487	81.8
PROSTATE GLAND	Adenocarcinoma	80 137	63.3
BREAST (FEMALE)	Invasive ductal carcinoma of female breast	79 381	62.7
LUNG	Adenocarcinoma, usual type	56 966	45.0
LIVER AND INTRAHEPATIC BILE TRACT	Hepatocellular carcinoma	32 847	26.0
RECTUM	Adenocarcinoma, usual type	31 522	24.9
OESOPHAGUS	Squamous cell carcinoma	21 958	17.3
LUNG	Squamous cell carcinoma	21 699	17.1
PANCREAS	Adenocarcinoma, usual type	21 053	16.6
BLADDER	Urothelial carcinoma	19 364	15.3
THYROID GLAND	Thyroid carcinoma, usual type	15 124	11.9
KIDNEY	Renal cell carcinoma, usual type	14 704	11.6
GALLBLADDER and EXTRAHEPATIC BILIARY TRACT	Adenocarcinoma, usual type	14 256	11.3
CORPUS UTERI	Adenocarcinoma, usual type	13 299	10.5
SKIN AND ADNEXAL	Basal cell carcinoma	12 896	10.2
LUNG	Neuroendocrine carcinoma, NOS	10 925	8.6
ORAL CAVITY AND LIP	Squamous cell carcinoma	9550	7.5
SKIN	Squamous cell carcinoma	8120	6.4

The site of the tumors was unknown in 47 770 cases; thus, whether they could be classified as rare cancers could not be determined.

### 3.2 Comparison With RARECARENet

The RARECARENet classification identified 15.4% of all cancer cases as rare, which is lower than the proportion obtained by the NCRC. In total, 74 609 cases of tumor types were not included in the original RARECARENet list; therefore, determining whether they could be classified as rare cancers was not possible. When we examined individual cases, 69 351 cases/year (6.8%) had changes in the rare/non‐rare classification between RARECARENet and NCRC, among which 45 293 and 232 109 cases (4 years) labeled as rare and non‐rare by RARECARENet became non‐rare and rare by NCRC from 2016 to 2019, respectively. The main reasons for the discrepancy in the rare/non‐rare classification between the NCRC and the RARECARENet list, along with specific examples and actual case numbers for each, are provided in Table 4. Examples of rare cancers based on the NCRC labeled as non‐rare by the RARECARENet list include diffuse large B‐cell lymphoma (DLBCL) and thyroid cancers, such as undifferentiated/anaplastic carcinoma. Conversely, oral cavity and lip cancers were rare in the RARECARENet list but were included in the same topographic category and common to the NCRC. Lung “squamous cell carcinoma, keratinizing, NOS” was categorized into “large cell carcinoma” and defined as rare by the RARECARENet classification; however, it was in the category of “squamous cell carcinoma” and defined as common by the NCRC. “Squamous cell carcinoma of the uterine cervix” was labeled as rare in the NCRC (5.99 cases/100 000/year) but common in the RARECARENet list, with an incidence of > 6 cases/100 000/year. Supporting Information S3: Table S2 shows the differences between the NCRC and RARECARENet lists.

**Table 4:** Reasons for the discrepancy in the rare/non‐rare classification between the NCRC and the RARECARENet list, including specific examples and the actual case number for each.

Non‐rare in RARECARENet, but rare in NCRC		
Summarized reasons for change	Example	*N* of cases
Difference in classification scheme	DLBCL separated by sites	112 823
Difference in site categorization	Appendix separated from colon	5661
Difference in histological subtype categorization	“Carcinoma, undifferentiated, NOS(8020/3)” of colon was previously categorized as Adenocarcinoma, but is now classified as Undifferentiated carcinoma, NOS	34 917
Inclusion of new histological subtypes	Undifferentiated/anaplastic carcinoma of thyroid separated	51 794
Other	—	189

Rare in RARECARENet, but non‐rare in NCRC		
Summarized reasons for change	Examples	*N* of cases
Difference in classification scheme	Neuroendocrine tumors separated by site	2
Difference in site categorization	Overlapping lesion of tongue(C28) classfied from oropharynx to oral cavity	728
Difference in histological subtype categorization	“Squamous cell carcinoma, keratinizing NOS(8071/3)” of lung was previously categolized as Large cell carcinoma, but is now classified as Squamous cell carcinoma	19 025
Inclusion of new histological subtypes	“Papillary carcinoma, NOS(8050/3)” of colon was previously categolized as Squamous cell carcinoma, but is now classfied as Adenocarcinoma, usual type	16 967
Other		5204

*Note:* Cases with no applicable RARECARE classifications were excluded (*N* = 31 969).

Abbreviation: *DLBCL, diffuse large B‐cell lymphoma.

## 4 Discussion

We developed a new cancer classification system, the NCRC, that can be applied to ICD‐O‐3.2 codes and developed a list of rare cancers in Japan using the NCR. For the NCRC classification system, we adopted a solid hierarchical structure in which Tier 1 solely reflected tumor sites, such that the list could serve as a diagnostic framework to distinguish various minor cancers with a clinically well‐applicable structure. Tier 2 classified pathological histology, which grouped similar biological/genomic characteristics. “Tier 1‐Rare” cancers are defined as those that occur in the sites where cancers are not common, and “Tier 2‐Rare” cancers are tumors that are common at the Tier 1 level but have a rare histology at the Tier 2 level. The structure of the NCRC makes the classification system clear and easy to understand. Furthermore, the NCRC can be used for surveillance and research purposes and for policymaking to control rare cancers. We found that rare cancers constitute approximately 20.0% of all cancer cases in Japan; this percentage is higher than that based on the RARECARENet list (15.4%). The difference is partially because our new list is more disaggregated than the European list. Rare cancers constitute a significant proportion of the cancer burden in Japan and Europe [10, 11].

The new NCRC system has several new characteristics. First, the structure of the NCRC was assigned as Tier 1 based on the tumor location. The most frequent discrepancies of rare cancer between NCRC and RARECARENet were caused by differences in the classification scheme (Table 4). DLBCL was treated as an independent category in RARECARENet, but NCRC classifies all DLBCLs into the involved sites. Histologically, tumors that can arise in many places throughout the body, such as lymphomas and sarcomas, were first classified according to their anatomical sites. We maintained this structure not only for the sake of consistency but also because patients’ initial contact with healthcare is usually based on the locations where they feel the abnormality and in what specialty they enter the clinical treatment flow. This led to the main difference from the RARECARENet classification, resulting in DLBCL in the extranodal location and lymph nodes being labeled as rare. Furthermore, NCRC Tier 1 was designed to be more consistent with the anatomical site classifications in the 8th edition of the Union for International Cancer Control Tumor‐Node‐Metastasis classification [12] and American Joint Commission on Cancer classification systems [13]. Therefore, although oral and lip cancers are separated and the larynx and hypopharynx are combined in RARECARENet, the NCRC places the oral and lip together and separates the larynx and hypopharynx. The site categorization also led to discrepancies, such as the separation of the appendix from the colon in the NCRC.

Second, the NCRC incorporates recent advances in terminology and pathohistological concepts introduced in the 5th edition of the WHO Classification of Tumors. For example, “spindle cell carcinoma” was classified as “sarcomatoid carcinoma” in the RARECARENet list. In contrast, the NCRC list categorized it as “carcinoma, NOS” because spindle cell carcinoma develops as a subset of either squamous cell or sarcomatoid carcinoma. According to the 5th edition of the WHO Classification of Tumors, hypopharyngeal, laryngeal, and tracheal spindle cell carcinoma are now classified as “spindle cell squamous cell carcinoma,” whereas lung spindle cell carcinoma is a subtype of “sarcomatoid carcinoma.” The term “spindle cell carcinoma” alone is not sufficient to differentiate two biologically distinct subsets; thus, the NCRC list classified “spindle cell carcinoma” as “carcinoma, NOS.” Similarly, “squamous cell carcinoma, non‐keratinizing” has been changed to squamous cell carcinoma from “large cell carcinoma” since the WHO classification 4th edition in 2015; however, this type remained in large cell carcinoma in RARECARENet. The use of the new ICD‐O 3.2 codes also enabled the capture of cases that were not classified by the RARECARENet system, owing to the lack of code allocation in the older ICD‐O‐3. The NCRC also included recently defined tumors and molecular alteration‐defined tumors. These tumors are well exemplified by hematolymphoid and CNS tumors, such as acute myeloid leukemia with t(8;21)(q22;q22.1); RUNX1‐RUNX1T1, myeloid/lymphoid neoplasms with PDGFRA rearrangement, diffuse midline glioma, H3 K27‐altered, CNS tumor with BCOR internal tandem duplication, and so on. As the landscape of genetically defined tumors continues to expand, considering these novel cancer categories and updating the list of rare cancers is important.

Third, the new histological classifications for several cancer sites were more detailed and divided into the most common histology of that site as non‐rare and the rest as rare. One example is the thyroid gland. RARECARENet classifies thyroid cancer as Tier 2, and the histology was divided only at Tier 3 level; however, even with this classification, thyroid cancer is judged as rare in Europe. When this classification is applied to Japanese populations, in which thyroid cancer is relatively common, the frequency exceeds the 6/100 000 threshold and is deemed not rare; nevertheless, undifferentiated thyroid cancer is a rare, distinct subtype of thyroid cancer clinically, and the NCRC system appropriately distinguishes and labels it as rare. Another example is epithelial tumors of peritoneal cancers; RARECARENet classifies only peritoneal mesothelioma and sarcoma of the peritoneum as epithelial tumors of peritoneal cancers owing to its tier structure. Tumors of the peritoneum are defined only in Tier 2 under the Tier 1 groups of “malignant mesothelioma” and “soft tissue sarcoma.” As the NCRC system categorizes tumors of the peritoneum at Tier 1, “adenocarcinoma” is included in Tier 2 as a subtype of “tumors of the peritoneum.”

Fourth, we excluded in situ diseases from the calculation of incidence. Although counting only invasive diseases in the incidence is a common practice and is adopted by RARECARENet, it has resulted in squamous cell carcinoma of the uterine cervix being labeled as rare. Because the number of in situ diseases is twice that of invasive diseases in Japan [14], clinicians who treat cervical cancer may feel odd about labeling cervical uterine cancer as rare. The rarity of invasive diseases of the uterine cervix is worth noting. Furthermore, when we applied the RARECARENet classification to the NCR, squamous cell carcinoma of the uterine cervix was not rare because its incidence was > 6/100 000/year. This difference was caused by glassy cell carcinoma (8015/3) and lymphoepithelial carcinoma (8082/3), both in the squamous cell carcinoma category in RARECARENet. However, as glassy cell carcinoma is considered a subtype of adenosquamous cell carcinoma, in the NCRC list, this subtype is in the category of a special type of adenocarcinoma rather than squamous cell carcinoma. Regarding lymphoepithelioma‐like carcinoma, as trans‐organ cancers are categorized as site‐agnostic tumors in the NCRC list, this tumor was listed separately from cervical tumors.

This study had some limitations. First, we developed a list of rare cancers by reflecting cases recorded in the NCR from 2016 to 2019 and the HBCR from 2016 to 2022. Histologies that were not present in these data were not classified. We must keep updating the classifications over several years. Second, the proposal of new cancer categories based on biomarker tests (e.g., “microsatellite instability‐high cancer,” “NTRK fusion cancer” [15, 16]) has become more common, which can significantly influence treatment options. Although our NCRC list is based solely on histological classification, integrating clinical insights or treatment methods according to biomarker test results is expected to be a challenging issue in the future. Third, the NCRC differentiates cancer entities to the extent that different codes are assigned in ICD‐O‐3.2. Some codes of ICD‐O‐3.2 group multiple cancer sites into the same code. For example, the topographic code (C24.0) is “extrahepatic bile duct,” which includes “cystic bile duct” in ICD‐O‐3.2. The “cystic bile duct” is usually grouped into “gallbladder (C23.9)” according to the classification of biliary tract carcinoma in Japan [17]; however, in this study, we put “cystic bile duct” into “extrahepatic bile duct” because we cannot discern “cystic bile duct” among “gallbladder (C23.9).” In another example, the topographic code (C44.2) is “external ear,” which includes “auricle” and “ceruminal gland” in ICD‐O‐3.2. Therefore, the NCRC cannot differentiate “external ear cancer,” which is usually treated by otolaryngologists, from “auricle cancer,” which is usually treated by dermatologists [18]. Fourth, we used the same criteria of annual incidence, 6/100 000, as the cut‐off to determine rare cancers. Because we have developed a new classification system, it may be worth reconsidering a different cut‐off in the future. Finally, our classification of rare cases is only as accurate as the accuracy of the cancer registrations, which depends on the clear specification of registration rules and educational support for the cancer registrars. To ensure accurate statistics and classifications, continuous efforts must be made to make the rules clinically relevant and to support registrars.

In conclusion, we developed a list of rare cancers in Japan using a classification list applicable to ICD‐O‐3.2 codes. We incorporated pathological concepts by adopting a new coding system and caught up with up‐to‐date clinical practice. The NCRC classification allows us to re‐examine the definition of rare cancers in Japan. Furthermore, it can also be applied to the definition of other cancers that may serve as policy targets, which is expected to facilitate the development of policies that capture the unique characteristics of each cancer. Furthermore, the NCRC can address the differences in the prevalence of tumors that are suggested to be more frequent in Asians through several ongoing collaborative studies with Southeast Asian countries, such as the MASTER Key Asia project [19]. We believe that incorporating ideas and data from diverse parts of the world will improve the standard reference for rare cancers and be useful for tailoring appropriate public healthcare systems worldwide.

## Author Contributions

Conception and design of the study: Akira Kawai and Takahiro Higashi. Methodology: Ryoko Rikitake, Yasushi Yatabe, and Takahiro Higashi. Software: Ryoko Rikitake, Yoko Yamamoto, and Takahiro Higashi. Validation: Ryoko Rikitake, Yasushi Yatabe, Yoko Yamamoto, and Takahiro Higashi. Acquisition and analysis of data: Ryoko Rikitake, Yasushi Yatabe, Yoko Yamamoto, and Takahiro Higashi. Investigation: Ryoko Rikitake, Yu Mizushima, and Takahiro Higashi. Resources: Ryoko Rikitake, Yasushi Yatabe, and Takahiro Higashi. Data curation: Ryoko Rikitake, Yasushi Yatabe, Tatsunori Shimoi, Shintaro Iwata, Yasushi Goto, and Takahiro Higashi. Drafting the manuscript or figures: Ryoko Rikitake, Yu Mizushima, and Takahiro Higashi. Writing‐review and editing: Ryoko Rikitake, Yasushi Yatabe, Tatsunori Shimoi, Shintaro Iwata, Yasushi Goto, Yu Mizushima, and Takahiro Higashi. Visualization: Ryoko Rikitake, Yu Mizushima, and Takahiro Higashi. Supervision: Akira Kawai. Project administration: Takahiro Higashi. Funding acquisition: Akira Kawai.

## Ethics Statement

In accordance with the procedure stipulated in the Cancer Registry Act, the protocol for NCR analysis was reviewed by the Data Utilization Committee of the National Cancer Registry Office. The authors are solely responsible for the analysis and interpretation of the extracted data and findings from the NCR database. As we followed the legal process for data acquisition and handling, the human subject research ethics guidelines in Japan exempt us from institutional ethics review. The use of HBCR was approved by the Institutional Review Board of the National Cancer Center and the Faculty of Medicine and Graduate School of Medicine of the University of Tokyo (2023343NI).

## Consent

The authors have nothing to report.

## Conflicts of Interest

Yatabe Yasushi is an editorial board member of Pathology International. The remaining authors declare no conflicts of interest.

## Supporting information

Supplementary Table S1. The NCRC. A: morphologic code list; B: topographic code list 3.1.

Supple tableS1B.

Supplementary Table S2. List of differences between the NCRC and RARECARENet list.

## Data Availability

The authors have nothing to report.
